# TLR4 contributes to the damage of cartilage and subchondral bone in discectomy‐induced TMJOA mice

**DOI:** 10.1111/jcmm.15763

**Published:** 2020-09-11

**Authors:** Xin Liu, Heng‐xing Cai, Pin‐yin Cao, Yaping Feng, Heng‐hua Jiang, Li Liu, Jin Ke, Xing Long

**Affiliations:** ^1^ The State Key Laboratory Breeding Base of Basic Science of Stomatology (Hubei‐MOST) & Key Laboratory of Oral Biomedicine Ministry of Education School & Hospital of Stomatology Wuhan University Wuhan China; ^2^ State Key Laboratory of Oral Diseases & National Clinical Research Center for Oral Diseases & Department of Orthognathic and TMJ Surgery West China Hospital of Stomatology Sichuan University Chengdu China; ^3^ Department of Oral and Maxillofacial Surgery School and Hospital of Stomatology Wuhan University Wuhan China

**Keywords:** cartilage, discectomy, osteoarthritis, subchondral bone, temporomandibular joint, TLR4

## Abstract

The abundance of inflammatory mediators in injured joint indicates innate immune reactions activated during temporomandibular joint osteoarthritis (TMJOA) progression. Toll‐like receptor 4 (TLR4) can mediate innate immune reaction. Herein, we aimed to investigate the expression profile and effect of TLR4 in the cartilage and subchondral bone of the discectomy‐induced TMJOA mice. The expression of TLR4 and NFκB p65 in the synovium of TMJOA patients was measured by immunohistochemistry, Western blotting and RT‐PCR. H&E and Masson staining were utilized to assess the damage of cartilage and subchondral bone of the discectomy‐induced TMJOA mice. A TLR4 inhibitor, TAK‐242, was used to assess the effect of TLR4 in the cartilage and subchondral bone of the discectomy‐induced TMJOA mice by Safranin O, micro‐CT, immunofluorescence and immunohistochemistry. Western blotting was used to quantify the expression and effect of TLR4 in IL‐1β–induced chondrocytes. The expression of TLR4 and NFκB p65 was elevated in the synovium of TMJOA patients, compared with the normal synovium. TLR4 elevated in the damaged cartilage and subchondral bone of discectomy‐induced TMJOA mice, and the rate of TLR4 expressing chondrocytes positively correlated with OA score. Intraperitoneal injections of TAK‐242 ameliorate the extent of TMJOA. Furthermore, TLR4 promotes the expression of MyD88/NFκB, pro‐inflammatory and catabolic mediators in cartilage of discectomy‐induced TMJOA. Besides, TLR4 participates in the production of MyD88/NFκB, pro‐inflammatory and catabolic mediators in IL‐1β–induced chondrocytes. TLR4 contributes to the damage of cartilage and subchondral bone in discectomy‐induced TMJOA mice through activation of MyD88/NFκB and release of pro‐inflammatory and catabolic mediators.

## 1 INTRODUCTION

Limited mouth opening, joint pain and noise are the primary performance of temporomandibular joint osteoarthritis (TMJOA) in clinical, affecting the living quality of patients greatly.^1^ Although the aetiology of TMJOA remains unknown, excessive loading on joint, such as masticatory muscle overload, malocclusion and jaw asymmetry, has been recognized as one of the main causes.^2^, ^3^, ^4^, ^5^, ^6^

Toll‐like receptor 4 (TLR4) is a pattern recognition receptor (PRR) which lies on the cell membrane and can mediate innate immune reaction.^7^ It is confirmed that the production of endogenous ligands induced by consistent burden or press strain on intervertebral disc cells, chondrocytes, kidney and heart activates TLR4 and promotes secretion of inflammatory mediators.^8^, ^9^, ^10^, ^11^ Moreover, up‐regulated TLR2 and TLR4 were detected in articular cartilage lesions and synovium of knee OA.^12^, ^13^ Meanwhile, elevated expression of TLR2, TLR3, TLR4 and TLR5 was confirmed in human OA cartilage compared with normal cartilage, verifying the role of Toll‐like receptors (TLRs) in the innate immune reactions during OA progression.^14^ By means of the pathological examination, a couple of inflammatory mediators, including IL‐1β, TNF‐α and IL‐6, have been detected in the tissues of TMJOA.^15^ Although frequently appears in bacterial inflammation, including pneumonia, periodontitis and enteritis, the flooding of inflammatory mediators in injured joint indicates innate immune reactions are involved in the development of TMJOA progression.^16^, ^17^, ^18^ Tissue damage and chronic inflammation both accounted for the activation of innate immune reactions during knee OA and TMJOA, while compared with the hyaline cartilages of knee joints, fibrocartilages in TMJ condyles display different characteristics regarding pressure‐bearing capacities, nutrition supply and constituent.^19^ Concerning that TLR4 can initiate immune reactions during the process of long‐term burden, it is reasonable to explore the effect of TLR4 in TMJOA, which has been characterized by synovitis, condylar degeneration and osteophyte. Indeed, recent studies reported that TLR4 elevated in the synovial membrane of temporomandibular joint (TMJ) synovitis induced by occlusal interference, and inhibition of TLR4 by TMJ injection of TAK‐242 could alleviate the level of synovitis, suggesting TLR4 participates in TMJ synovitis.^20^, ^21^

Animal model is an efficacious approach to investigate the pathology and progression of TMJOA as it is difficult to obtain clinical samples.^15^ Discectomy has been accepted as an effective strategy to mimic overloading in the TMJ because discs are responsible for dispersing intra‐articular pressure,^22^, ^23^ and it is easy to manipulate and duplicate.^24^, ^25^

Herein, this study was aimed to detect the alteration of TLR4 in the synovium of TMJOA patients, and to investigate the expression profile of TLR4, its downstream pathways, pro‐inflammatory and catabolic mediators in the cartilage as well as subchondral bone of the discectomy‐induced TMJOA mice. Furthermore, a specific TLR4 inhibitor was utilized to investigate the effect of TLR4 in the progression of TMJOA both in vivo and in vitro.

## 2 MATERIALS AND METHODS

### 2.1 Clinical samples collection

Two types of synovium of ‘OA’ groups and ‘normal’ groups were collected from patients undergoing arthroplasty, respectively. Synovial membrane samples of ‘OA’ groups were obtained from 10 patients (19‐62 years, average 31) suffering TMJOA accompanied with disc perforation, and synovial membrane samples of ‘normal’ groups verified noninflammatory by pathological diagnosis according to Murakami's criteria^26^ were acquired from 8 patients (20‐58 years, average 29) sustaining condylar hypertrophy. Patients complaining with other joint diseases or pretreated with medication were excluded. Informed consents were obtained before surgeries, and the protocol was approved by the Human Research Ethics Committee, School and Hospital of Stomatology, Wuhan University (protocol No. 2014LUNSHENZI24).

### 2.2 Discectomy‐induced TMJOA mice

The discectomy‐induced TMJOA model was employed in this study. Fifty‐four male C57BL/6J mice (8 weeks old) from the Experimental Animal Centre of Hubei Province were utilized. Three groups (discectomy, discectomy with TAK, and control) were established, and each was examined for three time points of 2, 4, and 6 weeks (n = 6). For the discectomy groups, microsurgery was performed by unilateral discectomy of right TMJ according to Lan's method.^24^ 10 mg/kg^27^ of TAK‐242 (HY‐11109, MCE, NJ, USA), a specific TLR4 inhibitor interfering with the interactions between TLR4 and its intracellular adaptors,^28^, ^29^ was administered by an intraperitoneal injection before the discectomy and maintained twice a week post‐surgery for the discectomy with TAK groups. Mice without any treatment were utilized as control. At 2, 4 or 6 weeks post‐surgery, the mice in each group were killed, respectively (n = 6). All the procedures of animal experiment were subjected to approval by the Ethics Committee for Animal Research, School and Hospital of Stomatology, Wuhan University, China (protocol No. 00273385).

### 2.3 Micro‐CT analysis

Temporomandibular joint tissues in each group were collected and fixed with 4% paraformaldehyde solution for 24 hours. After flushing overnight, TMJ tissues collected at week 6 post‐surgery were scanned by Micro‐CT (filter Al 0.2 mm, 50 kV, 500 µA, 12.59 μm, SkyScan1176) to record the change of subchondral bone, then reconstructed with NRecon (dynamic image range 0.019000‐0.050000) and analysed by CTAn (grey‐level threshold 120‐255) for the parameters including the ratio of bone volume to tissue volume (BV/TV), trabecular thickness (Tb. Th), trabecular number (Tb. N) and trabecular space (Tb. Sp). 3D images were reconstructed for morphological assessment.

### 2.4 Histological analysis

Temporomandibular joint tissues were demineralized in 10% ethylenediaminetetraacetic acid for 6 weeks and embedded in paraffin after gradient dehydration. Series mid‐sagittal sections of 5 μm were cut parallel to the condyle. After dewaxing in xylene and hydration in gradient alcohol, H&E, Safranin O and Masson trichrome staining were carried out according to the manufacturer's protocol. Cartilage thickness was determined from H&E staining on the basis of the predecessors' methods.^30^ A modified Mankin scoring system was used for cartilage OA score, which contains 4 criteria: pericellular Safranin O staining, background Safranin O staining, arrangement of chondrocytes, and cartilage structure.^24^, ^31^ Proteoglycan changes in cartilage matrix were detected using Safranin O staining. Masson trichrome staining was utilized to observe the change of subchondral bone and the degree of bone demineralization. Three regions of each section (anterior, middle and posterior of cartilage) were counted and averaged for cartilage thickness and OA score.

### 2.5 Immunohistochemistry

After dewaxing and hydration, the tissue sections were microwave antigen‐retrieved in citrate solution. For immunohistochemistry, the sections were endogenous peroxidase activity blocked, following by serum blocking unspecific ligations, rabbit anti‐TLR4 (1:400, 19811‐1‐AP, Proteintech), rabbit anti‐NFκB p65 (1:400, 10745‐1‐AP, Proteintech), rabbit anti‐IL‐1β (1:300, ab9722, Abcam), rabbit anti‐TNF‐α (1:300, ab6671, Abcam), rabbit anti‐ADAMTS5 (1:300, ab41037, Abcam) and rabbit anti‐MMP13 (1:300, 18165‐1‐AP, Proteintech) primary antibody were incubated overnight at 4°C severally, then reacted with anti‐rabbit UltraSensitive S‐P kit (KIT‐9706, Maixin) according to manufacturer's protocol. For calculating the rate of positive cells, Image‐Pro Plus 6.0 software was used to quantify the number of positive cells and total cells on selected cartilage area. Anterior, middle and posterior, 3 fields of each section were counted and averaged by two observers independently.

### 2.6 Immunofluorescence

For immunofluorescence, the antigen‐retrieved tissue sections were unspecific ligations blocked by serum after antigen‐retrieving. With rabbit anti‐TLR4 (1:300), rabbit anti‐NFκB p65 (1:300), and rabbit anti‐MyD88 (1:400, 23230‐1‐AP, Proteintech) primary antibody incubated overnight at 4°C, the sections were then reacted with fluorescence secondary antibodies, and nuclei stained by DAPI. Three fields of each section were used to record the rate of positive cells by two observers independently.

### 2.7 RT‐PCR

After total mRNA of clinical samples was extracted using TRIzol Kit (Invitrogen) and reversely transcribed, polymerase chain reaction was manipulated to cDNA, with the SYBR Premix Ex Taq (TaKaRa). Then, gene expression of mRNA was calculated using the cycle threshold method (2^−ΔΔCt^). The primer sequences were as follows: TLR4 (forward: CAAGAACCTGGACCTGAGCTTTA, reverse: GATTTGTCTCCACAGCCACCAG); NFκB p65 (forward: TCACCGGATTGAGGAGAAACG, reverse: CAGGGATGACGTAAAGGGATAGG); GAPDH (forward: GGAAGCTTGTCATCAATGGAAATC, reverse: TGATGACCCTTTTGGCTCCC). The gene of GAPDH was used as an internal control.

### 2.8 Chondrocytes culture

Forty male Sprague Dawley rats (4 weeks old) were used for TMJ chondrocytes harvesting in the present study. After digested with 0.25% trypsin for 20 minutes, TMJ cartilage was then digested with 0.1% collagenase type I combined with 0.1% collagenase type II for 1 hours. Digested chondrocytes were resuspended in DMEM containing 20% foetal bovine serum (FBS) after centrifugation and cultured in an atmosphere of 5% CO_2_ eventually. The chondrocytes of the second passage were incubated with 10 ng/mL of IL1‐β (400‐01B, Proteintech) for 0, 6, 12, 24, 36 and 48 hours severally or IL‐1β (0, 2, 5, 10, 50 and 100 ng/mL, respectively) for 24 hours. To investigate the role of TLR4 in IL‐1β–induced expression of pro‐inflammatory and catabolic mediators in chondrocytes, cells were pre‐treated with 10 μmol/L^32^, ^33^ TAK‐242 for 3 hours, and then, 10 ng/mL of IL‐1β was added and treated for 24 hours.

### 2.9 Western blotting

The collected and measured proteins were separated by SDS‐PAGE electrophoresis and then electro‐transferred onto a PVDF membrane. After blocking with non‐fat milk, the membrane was incubated with different primary antibodies at 4°C overnight, respectively, including rabbit anti‐TLR4 (1:1000), rabbit anti‐MyD88 (1:2000), rabbit anti‐NFκB p65 (1:1000), rabbit anti‐TNF‐α (1:1000), rabbit anti‐COX‐2 (1:3000, 12375‐1‐AP, Proteintech), rabbit anti‐MMP13 (1:3000) and mouse anti‐MMP3 (1:10 000, 66338‐1‐Ig, Proteintech). Then, HRP‐conjugated secondary antibody was incubated with PVDF membrane, and a chemiluminescence ECL system (Advansta) was utilized to detect the immunoreactive proteins, as described previously.^34^

### 2.10 Statistical analysis

Statistical analysis was performed by Prism 8.0 (GraphPad software). A Shapiro‐Wilk test for normality was utilized to determine a suitable test for parametric or non‐parametric populations, with *F*‐tests performed to determine whether equal variance of populations. A Student *t* test was used when the variables demonstrated parametric distributions; otherwise, non‐parametric Mann‐Whitney *U* test was performed. *P* < .05 was considered significant statistically. All quantitative data were presented as the mean ± SEM.

## 3 RESULTS

### 3.1 Elevation of TLR4 and NFκB p65 in the synovium of TMJOA patients

**FIGURE 1:**
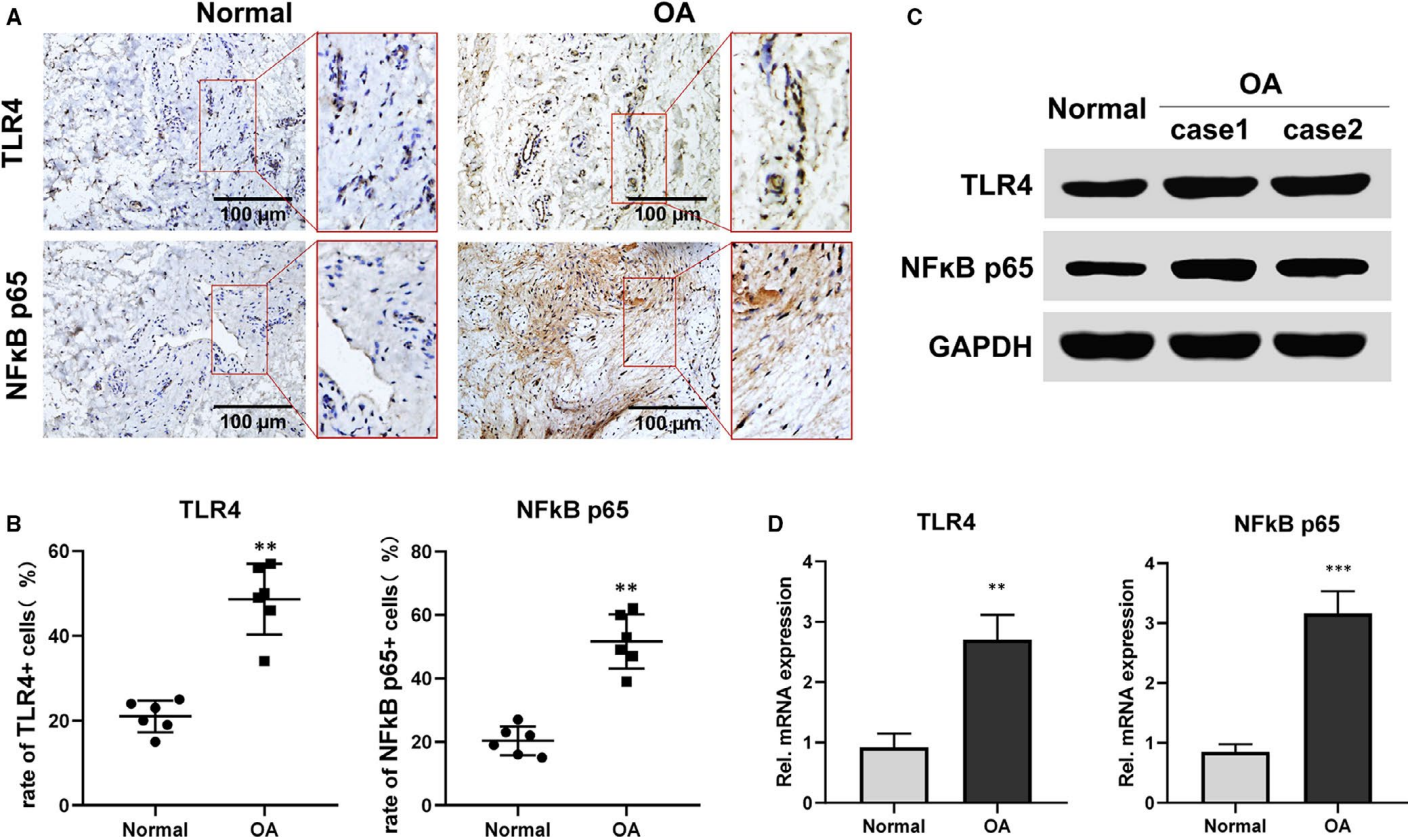
Elevation of TLR4 and NFκB p65 in the synovium of TMJOA patients. A and B, Representative sections of TMJOA or normal synovium were detected by immunohistochemistry (n = 6). TMJOA synovium showed significant and homogeneous positive staining of TLR4 and NFκB p65, while normal synovium exhibited inconspicuous staining (A). The rate of TLR4‐ or NFκB p65–positive cells increased in TMJOA groups compared with normal (B). C, Western blotting results exhibited that the bands of TLR4 and NFκB p65 of TMJOA synovium appeared thicker compared with those of normal. D, The expression of TLR4 or NFκB p65 gene was increased in the synovium of TMJOA compared with normal by RT‐PCR (n = 4). Data were analyzed by Mann‐Whitney *U* test (**P < .01, ***P < .001) (OA represents TMJOA)

Compared with the normal synovium, the expression of TLR4 and NFκB p65 was enhanced in the synovium of TMJOA patients detected by immunohistochemistry (Figure 1A), accompanied by the increased rate of TLR4‐ and NFκB p65–positive cells (Figure 1B). Western blotting results exhibited that the bands of TLR4 and NFκB p65 of TMJOA synovium appeared thicker compared with those of normal (Figure 1C). Similarly, the gene expression of TLR4 and NFκB p65 detected by RT‐PCR was significantly higher in TMJOA synovium, compared with that in normal (Figure 1D) (**P < .01, ***P < .001).

### 3.2 TLR4 accounts for the damage of cartilage and subchondral bone in discectomy‐induced TMJOA mice

**FIGURE 2:**
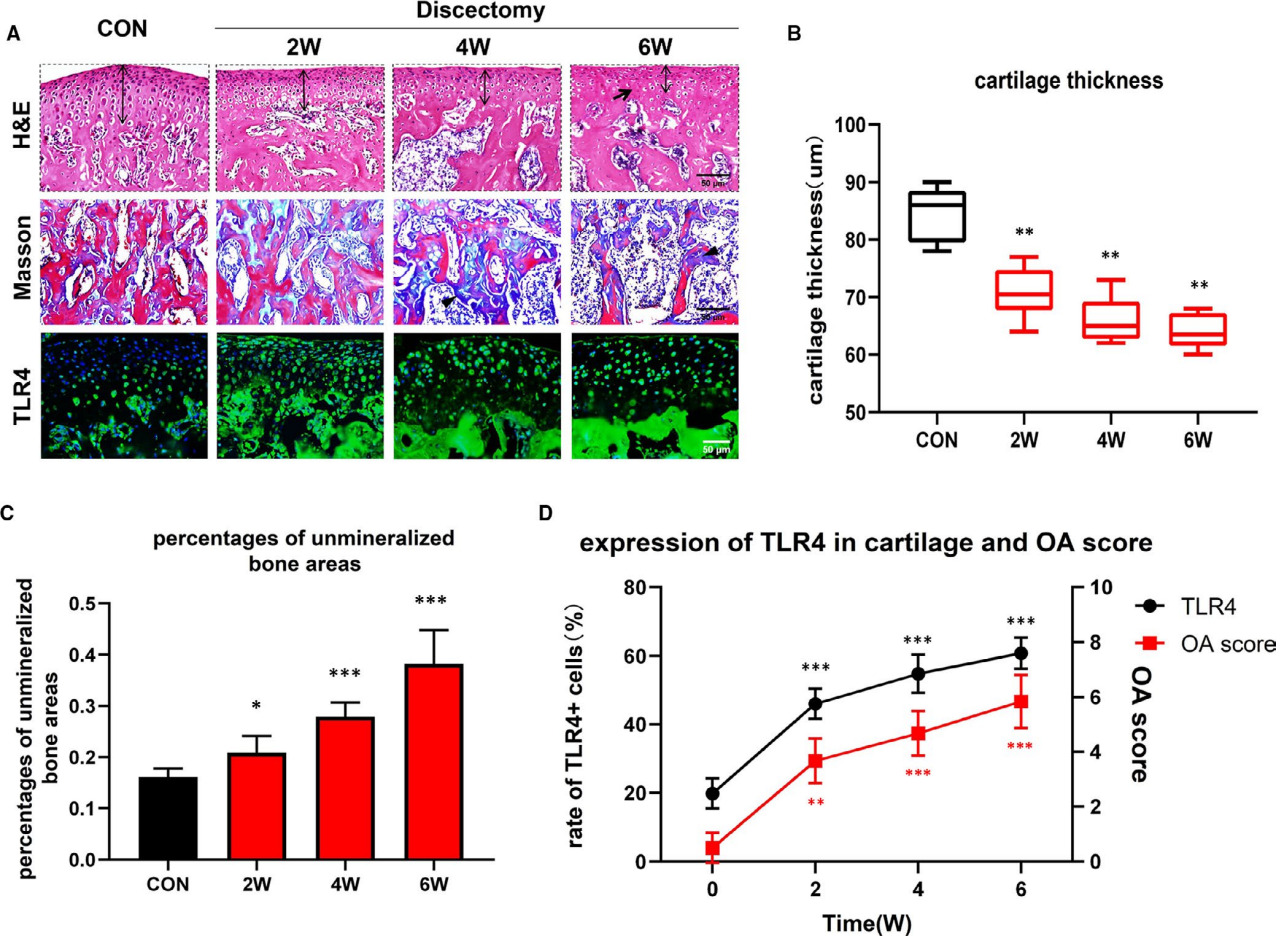
Elevation of TLR4 positively correlates with the damage of cartilage and subchondral bone in discectomy‐induced TMJOA mice. A, Compared with control, there was a time‐dependent cartilage thinning (two‐way arrow), chondrocytes decreasing (black arrow) and unmineralized bone areas increasing (black arrowhead), as well as elevation of TLR4 in the cartilage and subchondral bone of discectomy groups. B, Comparison between control and discectomy groups regarding cartilage thickness (*: vs CON, horizontal solid lines, medians; box plots, 25th and 75th percentiles; horizontal bars, minimum and maximum). C, Gradually increased the percentages of unmineralized bone areas in the subchondral bone of discectomy mice comparing with control mice (*: vs CON). D, The expression of TLR4 was increased in the cartilage of discectomy groups gradually. The rate of TLR4 expressing cells in the cartilage was positively correlated with OA score (*: vs 0W). (Data were calculated at each time point using Student's *t* test, n = 6, *P < .05, **P < .01, ***P < .001). (CON represents control; OA represents TMJOA)

As shown in Figure 2, the discectomy groups revealed a time‐dependent cartilage thinning, unmineralized bone areas enlarging and chondrocytes decreasing in condyle, comparing with control. Immunofluorescence results exhibited that TLR4 expression was elevated gradually in the cartilage and subchondral bone from the discectomy groups (Figure 2A). Moreover, the rate of TLR4 expressing cells in the cartilage was positively correlated with OA score (Figure 2D) (*P < .05, **P < .01, ***P < .001).

**FIGURE 3:**
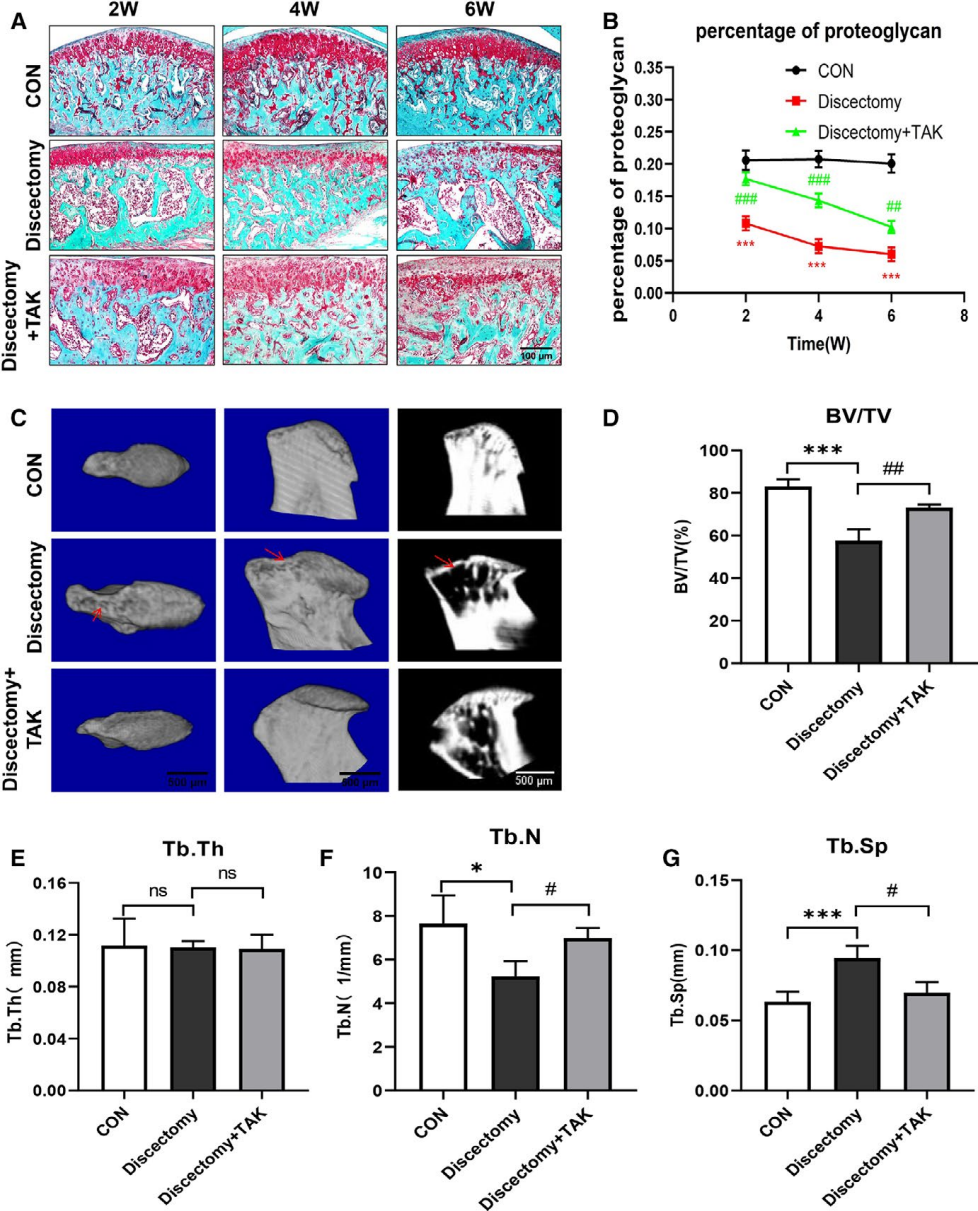
TLR4 participates in the damage of cartilage and subchondral bone in discectomy‐induced TMJOA mice. A, The arrangement of control cartilage was regular, and its matrix was abundant, in contrast, obvious disorder occurred in the cartilage of discectomy groups, while appeared mitigated after twice a week of TAK‐242 injections. B, The percentage of proteoglycan gradually decreased in the discectomy groups compared with control, whereas TAK‐242 injections significantly ameliorate the reducing of cartilage proteoglycans induced by discectomy alone (*: discectomy groups vs control; #: discectomy + TAK groups vs discectomy groups). C, Larger bone lesion of condyle (red arrows) and obvious atypical condyle shape were found in the micro‐CT image of discectomy groups, while these phenomena were ameliorated after twice a week of TAK‐242 injections. D‐G, The remodelling of subchondral bone was assessed by histomorphometric parameters, including ratio of bone volume to tissue volume (BV/TV), trabecular thickness (Tb. Th), trabecular number (Tb. N) and trabecular space (Tb. Sp). (Data were analysed by Student's *t* test, n = 3‐6, *P < .05, ***P < .001; #*P* < .05, ##*P* < .01, ###*P* < .001). (CON represents control; Discectomy + TAK represents discectomy combined with TAK‐242 injection groups)

Besides, to further explore the effect of TLR4 in the injured cartilage and subchondral bone of TMJOA, a specific TLR4 inhibitor, TAK‐242, was administered intraperitoneally before the discectomy and maintained twice a week in this study. By Safranin O staining, a time‐dependent reduction of proteoglycans occurred in the cartilage of discectomy groups as compared with that of control, which maintained abundant and regular proteoglycans (Figure 3A,B). Furthermore, TAK‐242 injections significantly ameliorate the lessening of cartilage proteoglycans induced by TMJ discectomy (Figure 3A,B). Based on micro‐CT analysis, there were obvious bone lesions and irregular surface at week 6 in the discectomy groups, whereas smaller lesions and improved appearance in the discectomy with TAK groups (Figure 3C). Similarly, quantitative evaluation results of micro‐CT including bone volume fraction (BV/TV), trabecular number (Tb.N) and trabecular separation (Tb.Sp) were consistent with morphological assessment, though there was no difference in trabecular thickness (Tb.Th) (Figure 3D‐G) (*P < .05, ***P < .001; #*P* < .05, ##*P* < .01, ###*P* < .001).

### 3.3 TLR4 promotes the expression of MyD88/NFκB, pro‐inflammatory and catabolic mediators in mice cartilage of discectomy‐induced TMJOA

**FIGURE 4:**
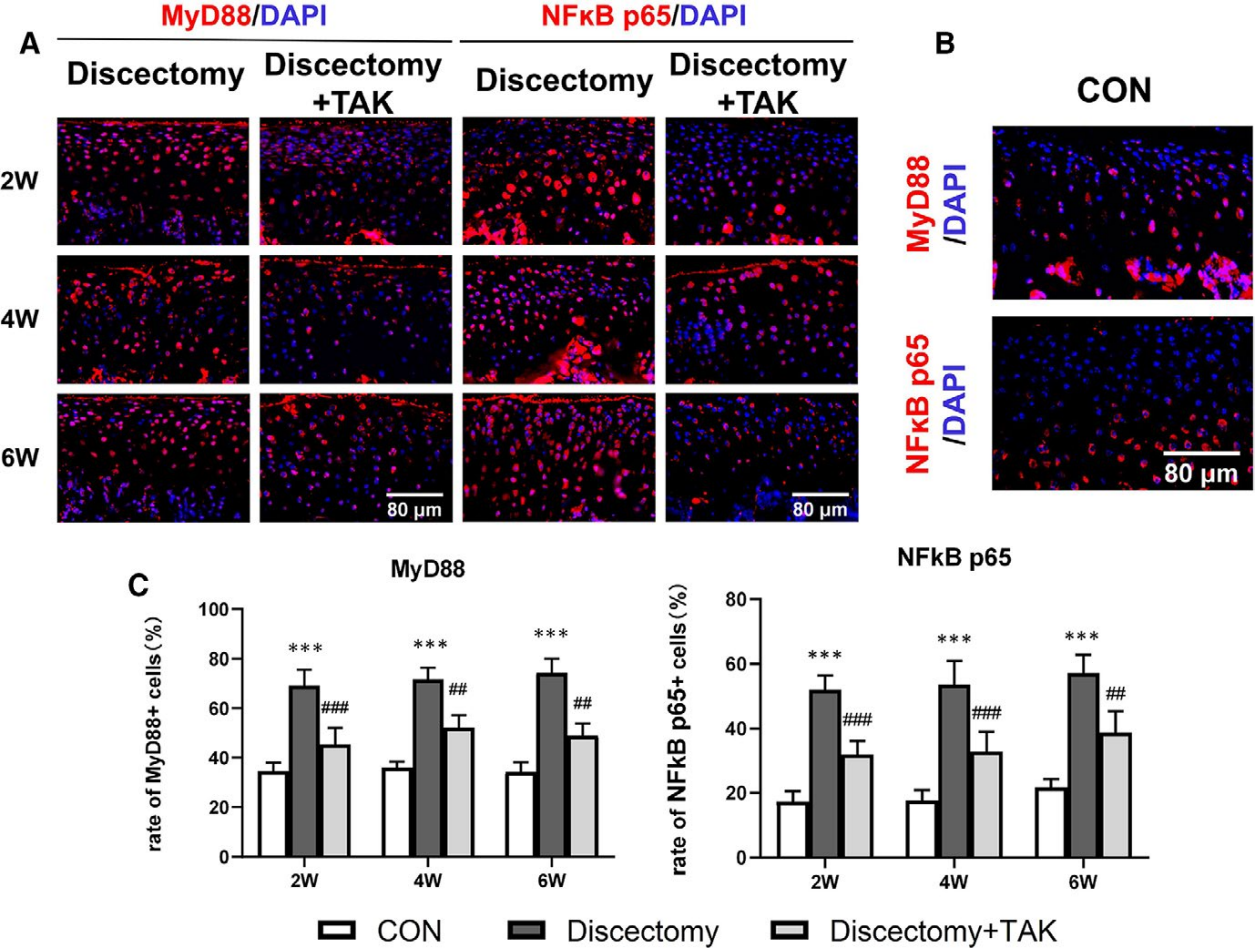
TLR4 induces the expression of MyD88/NFκB in discectomy‐induced TMJOA mice. A and B, The expression of MyD88 and NFκB p65 in the cartilage of discectomy‐induced TMJOA detected by immunofluorescence at 2, 4, 6 wk (A), or in the cartilage of control (B). C, The rate of MyD88‐ or NFκB p65–positive cells in the cartilage of discectomy‐induced TMJOA mice was significantly increased, while inhibited by TAK‐242 treatment (*: Discectomy groups vs control, #: Discectomy + TAK groups vs Discectomy groups; Data were analysed by Student's *t* test, n = 6, ***P < .001; ##*P* < .01, ###*P* < .001). (CON represents control; Discectomy + TAK represents discectomy combined with TAK‐242 injection groups)

Contrasting with control, the expression of MyD88 and NFκB p65 increased in the cartilage of discectomy groups at all time points, with more positive cells found in the fibre layer (Figure 4A,B). Likely, TAK‐242 treatment significantly decreased the expression of MyD88 and NFκB p65 in the damaged cartilage induced by discectomy (Figure 4A,C) (***P < .001; ##*P* < .01, ###*P* < .001).

**FIGURE 5:**
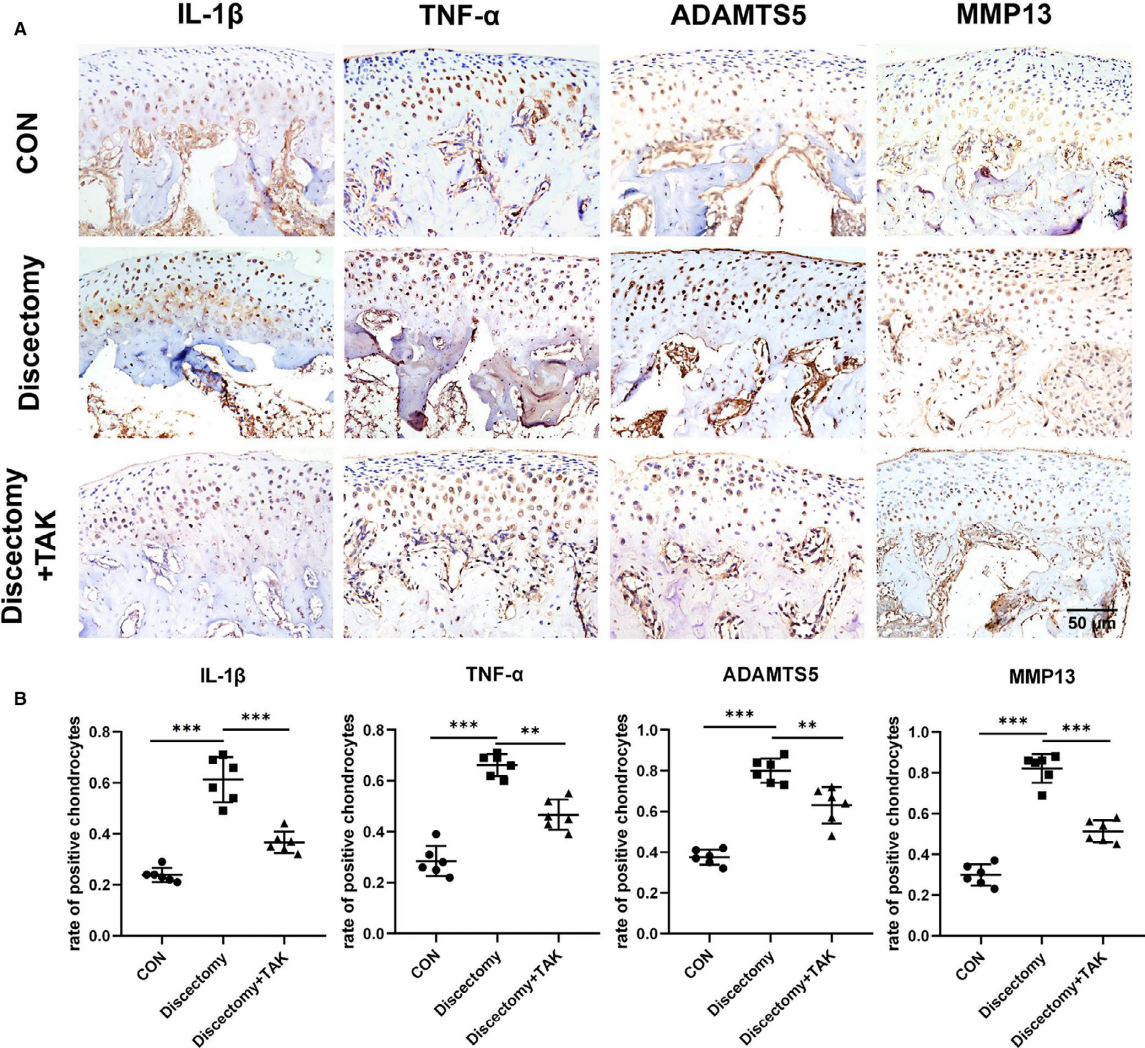
TLR4 promotes the expression of cartilage pro‐inflammatory and catabolic mediators in discectomy‐induced TMJOA mice. A, The expression of IL‐1β, TNF‐α, ADAMTS5 and MMP13 was detected by immunohistochemistry in the cartilage of discectomy groups, discectomy with TAK‐242 treatment groups at week 6, or in the control cartilage. B, The rate of IL‐1β, TNF‐α, ADAMTS5 or MMP13 positive cells in the cartilage was markedly increased in the discectomy groups, while markedly inhibited after treatment with TAK‐242. (Data were analysed by Student's *t* test, n = 6, **P < .01, ***P < .001). (CON represents control; Discectomy + TAK represents discectomy combined with TAK‐242 injection groups)

Additionally, the expression of pro‐inflammatory mediators (IL‐1β, TNF‐α) and catabolic mediators (ADAMTS5, MMP13) in cartilage was detected by immunohistochemistry. At week 6, the expression of IL‐1β, TNF‐α, ADAMTS5 and MMP13 in the discectomy groups increased when compared with that in control, while decreased in the discectomy with TAK groups (Figure 5A,B) (**P < .01, ***P < .001).

### 3.4 TLR4 participates in the production of MyD88/NFκB, pro‐inflammatory and catabolic mediators in IL‐1β induced chondrocytes

**FIGURE 6:**
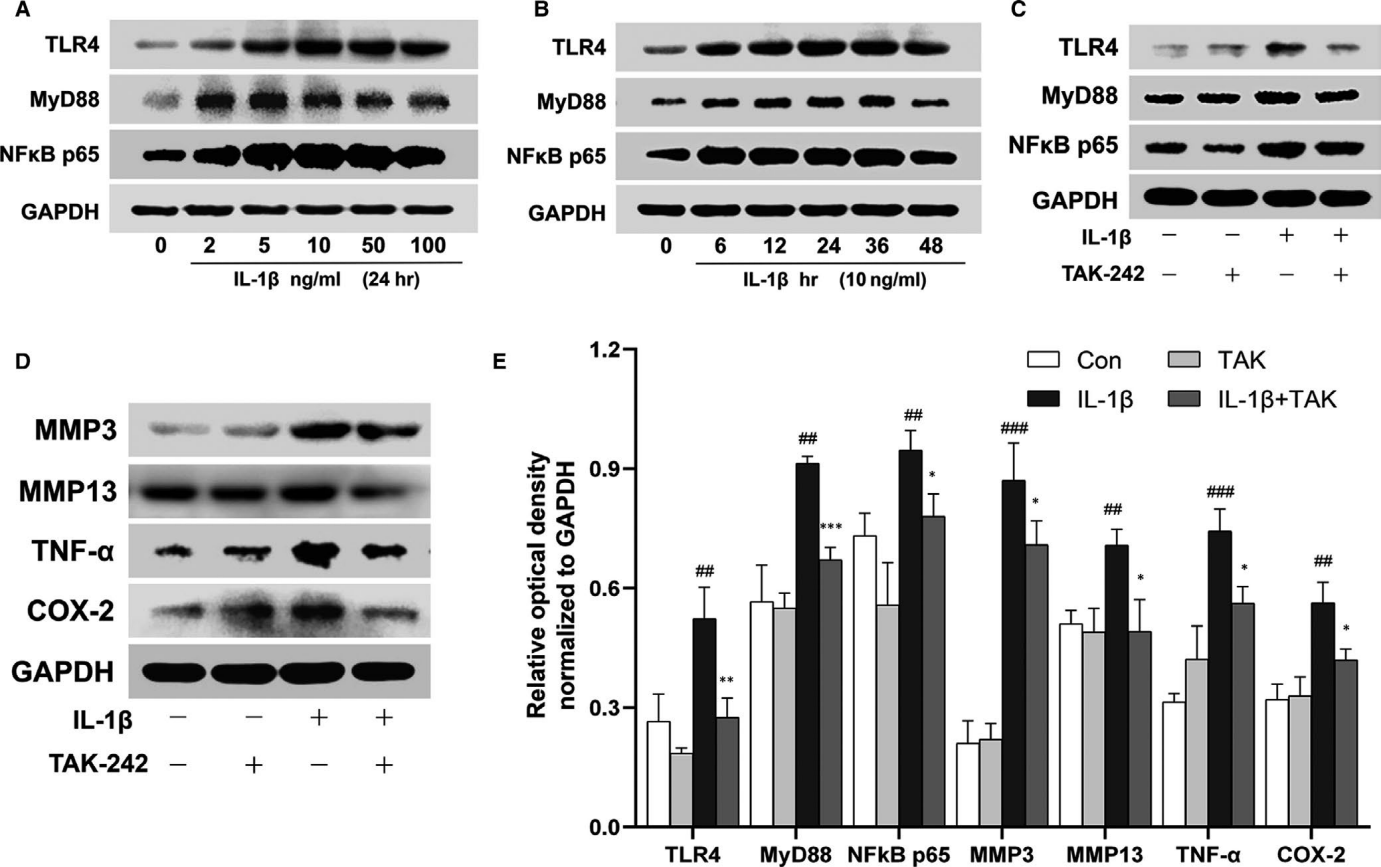
TLR4 participates in the production of MyD88/NFκB, pro‐inflammatory and catabolic mediators in IL‐1β–induced chondrocytes. A, A dose‐dependent augmentation of TLR4, MyD88 and NFκB p65 in rat chondrocytes was induced by IL‐1β for 24 h. B, A time‐dependent increase of TLR4, MyD88 and NFκB p65 in these cells was induced by 10 ng/mL of IL‐1β. C and D, Cells were pre‐treated with 10 μmol/L TAK‐242 for 3 h, and then, 10 ng/mL of IL‐1β was treated for 24 h. The production of TLR4, MyD88, NFκB p65, MMP3, MMP13, TNF‐α and COX‐2 was detected by Western blotting. (E) Proteins relative optical density normalized to GAPDH was calculated. IL‐1β promoted the production of pro‐inflammatory mediators (TNF‐α, COX‐2) and catabolic mediators (MMP3, MMP13) in chondrocytes, whereas pretreatment of TAK‐242 significantly reduced the production of MyD88/NFκB and above mediators induced by IL‐1β (#: IL‐1β group vs Con group, *: IL‐1β + TAK group vs IL‐1β group. Data were analysed by Mann‐Whitney U test, n = 3, ##*P* < .01, ###*P* < .001; *P < .05, **P < .01, ***P < .001)

A dose‐ and time‐dependent elevation of TLR4, MyD88 and NFκB p65 in rat chondrocytes induced by IL‐1β and 10 ng/mL of IL‐1β for 24‐h incubation is optimal (Figure 6A,B). Also, the production of pro‐inflammatory mediators (TNF‐α, COX‐2) and catabolic mediators (MMP3, MMP13) was up‐regulated in these cells induced by IL‐1β. Furthermore, pretreatment of TAK‐242 significantly reduced the production of MyD88/NFκB and the above mediators in these cells induced by IL‐1β (Figure 6C‐E) (##*P* < .01, ###*P* < .001; *P < .05, **P < .01, ***P < .001).

## 4 DISCUSSION

Activation of the innate immune system is involved in the initiation and perpetuation of tissue damage or chronic inflammation. As a classic innate immunity mediator, TLR4 has been currently found to be elevated in rat TMJ synovitis induced by occlusal interference, and synovitis mitigated by TMJ injection of TAK‐242.^20^ In ex vivo experiments of LPS incubated TMJ synovial fibroblasts, pretreatment of TAK‐242 could decline the elevated expression of inflammatory factors.^35^ In the present study, TLR4 was highly expressed in the synovium of TMJOA patients, compared with condylar hypertrophy patients' synovium, which has been identified as a noninflammatory normal synovial specimens by our histochemical professionals according to the criteria of Murakami.^26^ In terms of patients with condylar hypertrophy, mandible deviation is the striking feature in clinic, while these patients exhibit no pain and normal mouth opening, whose synovium has been verified and used as normal in our former study,^5^ as normal synovium cannot be acquired from normal human beings. Meanwhile, an augmented expression of TLR4 occurred in mouse cartilage and subchondral bone of discectomy‐induced TMJOA. During 6 weeks after the discectomy surgery, there was a time‐dependent cartilage thinning and unmineralized bone areas enlarging, as well as cartilage OA score increasing, which was consistent with predecessor's observation.^24^, ^36^ However, regarding the synovitis induced by discectomy, there is no obvious inflammation in the synovium based on our observation (data not shown), speculating two reasons for this phenomenon. One is that discectomy cannot jeopardize synovium, and the other is that synovial inflammation has vanished before samples were harvested at 2 weeks. Notably, cartilage OA score was positively correlated with the elevation of TLR4. Furthermore, when TAK‐242 injected intraperitoneally, the damage of mouse cartilage and subchondral bone induced by TMJ discectomy was remarkably attenuated. Therefore, these findings indicate that TLR4 contributes to synovitis, the degeneration of cartilage and breakdown of subchondral bone, which are key features of TMJOA.

Accompanied by the elevation of TLR4, the expression of NFκB p65 was also up‐regulated in the synovium of TMJOA patients. Former literatures reported that TLR4 binds to its corresponding ligands, followed by activating MyD88 and ultimately leading to the activation as well as nuclear translocation of the NFκB.^37^, ^38^ This prompted us to postulate that substantial TLR4 in the cartilage and subchondral bone of TMJOA mice might evoke the activation of NFκB to participate in the degeneration of those corresponding areas. Indeed, increased expression pattern of MyD88 and NFκB p65 was observed in mouse cartilage of discectomy‐induced TMJOA. In contrast, TAK‐242 significantly inhibited the expression of MyD88 and NFκB p65 in that area. Our previous study and others have demonstrated that activated NFκB, or elevation of NFκB p65, governs the production of a series of pro‐inflammatory and catabolic mediators in TMJ tissues,^39^, ^40^, ^41^, ^42^, ^43^ and the flooding of pro‐inflammatory and catabolic mediators exacerbates the degradation of condyle, thereby leading to joint damage.^44^ In the present study, pro‐inflammatory mediators (IL‐1β, TNF‐α) and catabolic mediators (ADAMTS5, MMP13) were significantly raised in mouse cartilage at 6 weeks after the discectomy surgery, while obviously decreased after administration of TAK‐242.

The abundance of IL‐1β in joint is a striking feature of TMJOA development.^15^, ^45^ Additionally, various joint‐derived cell studies, such as synovial fibroblasts, chondrocytes and osteoblasts studies as well, used IL‐1β as one of TMJOA stimulators.^46^, ^47^, ^48^, ^49^ Thus, we utilized IL‐1β stimulating chondrocytes to mimic TMJOA condition in vitro. A dose‐ and time‐dependent expression of TLR4, MyD88 and NFκB p65 in IL‐1β–induced chondrocytes was discovered, while the expression of pro‐inflammatory and catabolic mediators decreased, along with the down‐regulation of TLR4, MyD88 and NFκB p65 when TAK‐242 was added. Notably, though IL‐1β is not the ligand of TLR4, certain inflammatory candidates raised by IL‐1β can induce expression of TLR4 in chondrocytes. For instance, high mobility group box 1 (HMGB1), elevated in IL‐1β–incubated chondrocytes by former researchers,^50^, ^51^, ^52^ is speculated as one of TLR4 activators in our in vitro experiments. These findings further corroborate our in vivo results from discectomy‐induced TMJOA mice.

It should be noted that TAK‐242 administered by intraperitoneal injection can approach subchondral bone and synovium through blood transport. Undoubtedly, there is no vessel in normal cartilage. However, under the circumstance of TMJOA, obvious vascular invasion occurs in the damaged cartilage.^53^ Therefore, TAK242 can reach synovium, cartilage and subchondral bone of TMJOA mice by blood transport, and target against TLR4 effectively. Meanwhile, it is reasonable to assume that catabolic mediators from synovial fluid are lessened because TAK242 inhibits the production of TLR4 and the downstream inflammatory mediators in the synovium, thereby resulting in the attenuation of the damage of cartilage and subchondral bone. Therefore, the above two reasons can explain our in vivo experiments using intraperitoneal injection of TAK‐242.

In the present study, the elevation of TLR4 was accompanied by the augmentation of MyD88, NFκB p65 as well as pro‐inflammatory and catabolic mediators both in discectomy‐induced TMJOA mice and in IL‐1β–induced chondrocytes, and vice versa. Although these findings cannot illustrate the explicit mechanism that how TLR4 manipulates TMJOA progression, it is strongly indicated that TLR4 contributes to the damage of cartilage and subchondral bone during TMJOA progression through activation of MyD88/NFκB to release the pro‐inflammatory and catabolic mediators, as the role of TLR4/MyD88/NFκB signal pathway in regulating inflammatory mediators has been specified in varied diseases.^54^, ^55^, ^56^, ^57^

## CONFLICT OF INTEREST

The authors confirm that there are no conflicts of interest.

## AUTHOR CONTRIBUTION

**xin liu:** Conceptualization (equal); Methodology (lead); Software (equal); Writing‐original draft (equal). **Hengxing CAI:** Conceptualization (equal); Formal analysis (lead); Software (equal). **Pinyin Cao:** Conceptualization (equal); Methodology (equal); Software (lead). **yaping feng:** Formal analysis (equal). **henghua jiang:** Methodology (equal). **li liu:** Software (equal). **Jin KE:** Conceptualization (equal); Data curation (lead); Project administration (lead); Writing‐original draft (equal). **Xing Long:** Conceptualization (equal); Data curation (lead); Supervision (lead); Writing‐original draft (lead).

## Data Availability

The data supporting the findings of this study are available from the corresponding author upon reasonable request.
