# Correction to ‘TLR4 Contributes to the Damage of Cartilage and Subchondral Bone in Discectomy‐Induced TMJOA Mice’

**DOI:** 10.1111/jcmm.71263

**Published:** 2026-07-01

X. Liu , H. X. Cai , P. Y. Cao , et al., “TLR4 Contributes to the Damage of Cartilage and Subchondral Bone in Discectomy‐Induced TMJOA Mice,” Journal of Cellular and Molecular Medicine 24, no. 19 (2020): 11489–11499, 10.1111/jcmm.15763.32914937 PMC7576306

In the originally published version of this article, an error was identified in Figure 4A during a recent re‐examination of the original data.

In Figure 4A, within the panel for 6 weeks of MyD88 in the Discectomy + TAK group, an incorrect representative image was inadvertently used during final figure assembly. Specifically, the image that should have shown the 6‐week time point was mistakenly displayed by a 4‐week time point image from the same experimental group.

The corrected Figure 4A is provided below. This correction does not affect the experimental results, statistical analyses, conclusions.

**FIGURE 4:**
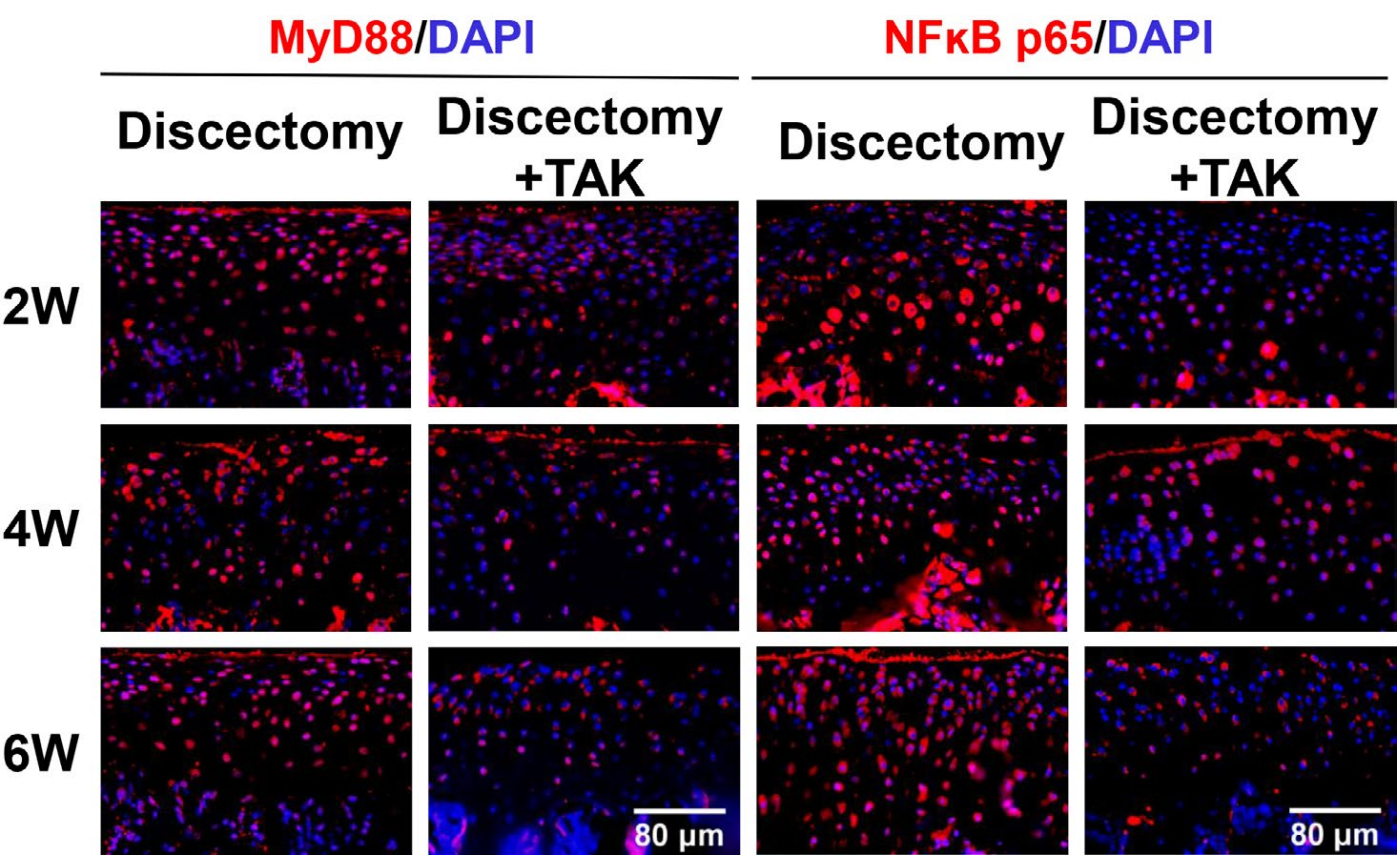
TLR4 induces the expression of MyD88/NFκB in discectomy‐induced TMJOA mice. (A, B) The expression of MyD88 and NFκB p65 in the cartilage of discectomy‐induced TMJOA detected by immunofluorescence at 2, 4, 6 weeks (A), or in the cartilage of control (B).

